# Notices

**Published:** 2009

## notice of duplicate publication

Madani TA. Hepatitis C virus infections reported in Saudi Arabia over 11 years of surveillance. Ann Saudi Med 2007;27:191-4. PMID: 17568177

It has been brought to our attention that this article subsequently appeared in substantially the same form in the *Transactions of the Royal Society of Tropical Medicine and Hygiene* (2009;103:132-136. PMID: 18789464). Submission of a previously published article or subsequent publication elsewhere of an article published in the *Annals*, without our knowledge, is a violation of our policy and international publication norms. This notice of duplicate publication will appear in MEDLINE. We have notified the author by email and courier mail but he did not reply nor provide any explanation for this duplicate publication We are following the recommendations of the Committee of Publication Ethics, who recommend that a notice of duplicate publication be published and the author's institution be notified. The author is banned from further publication in the *Annals of Saudi Medicine* for a period of 5 years.

## notice of triplicate publication

Tamiolakis D, Nicolaidou S, Bolioti S, Tzilivaki A. Prognostic significance of major histocompatibility complex class II antigens (HLA-DR) in normal colonic mucosa, tubulovillous adenoma, and invasive colonic carcinoma. Ann Saudi Med 2006;26:133-7. PMID: 16761451.

It has been brought to our attention that the above article substantially duplicated two other articles by several of the same authors, one published in the journal *Chirugia* (2005;100:451-456. PMID: 16372671) and the other in *Cesk Patol* (2006 Apr;42:52-8. PMID: 16715627). Submission of a previously published article or subsequent publication elsewhere of an article published in the *Annals*, without our knowledge, is a violation of our policy and international publication norms. This notice of duplicate publication will appear in MEDLINE. We are following the recommendations of the Committee of Publication Ethics, who recommend that a notice of duplicate publication be published and the author's institution be notified. At the time of submission to the *Annals*, the authors stated that the article had not been previously published or submitted elsewhere. All authors on the article that appeared in the *Annals* are banned from further publication in the *Annals of Saudi Medicine.*

